# Effect of Single Session of Anodal M1 Transcranial Direct Current Stimulation—TDCS—On Cortical Hemodynamic Activity: A Pilot Study in Fibromyalgia

**DOI:** 10.3390/brainsci12111569

**Published:** 2022-11-18

**Authors:** Marianna La Rocca, Livio Clemente, Eleonora Gentile, Katia Ricci, Marianna Delussi, Marina de Tommaso

**Affiliations:** 1 Physics Department, Bari Aldo Moro University, 70121 Bari, Italy; 2 Laboratory of Neuro Imaging, USC Stevens Neuroimaging and Informatics Institute, Keck School of Medicine of USC, University of Southern California, Los Angeles, CA 90007, USA; 3 DiBraiN Department, Bari Aldo Moro University, 70121 Bari, Italy

**Keywords:** fibromyalgia, motor networks, functional near infrared spectroscopy, transcranial direct current stimulation (TDCS)

## Abstract

Transcranial direct current stimulation (TDCS) on the primary motor cortex (M1) has been reported to be effective in fibromyalgia (FM). Our previous works have shown hypometabolism of motor networks in FM using Functional Near Infrared Spectroscopy (fNIRS), which could contribute to pain symptoms. To investigate if a single Transcranial Direct Current Stimulation (TDCS) session can restore the reduced metabolism expected in FM patients, we compared metabolic activity in FM patients and controls during a finger-tapping task in basal condition, sham condition, and under anodal TDCS on M1. During the finger tapping task, a continuous wave 20 channel fNIRS system was placed across the bilateral central-frontal areas in 22 healthy controls and 54 FM patients. Subjects were randomly assigned to real TDCS or sham stimulation. The finger-tapping slowness did not change after real and sham stimulation. After real TDCS stimulation, FM patients showed an increased activation of cortical motor regions (t-statistic = −2.5246, *p*-value = 0.0125 for the stimulated hemisphere and t-statistic = −4.6638, *p*-value = 0.0001 for the non-stimulated hemisphere). The basal differences between FM and controls reverted after real TDCS, while this effect was not observed for sham stimulation. A single TDCS session of the cortical motor network seemed able to restore basic cortical hypometabolism in FM patients. Further studies could clarify the long-term effect of M1 stimulation on cortical metabolism, and its relevance in pain processing and clinical features.

## 1. Introduction

Fibromyalgia (FM) is a complex syndrome characterized by generalized musculoskeletal pain, fatigue, muscle stiffness, sleep, affective disturbances, as well as cognitive problems. Fibromyalgia is currently underdiagnosed; its pathophysiology is not fully understood, and pharmacological treatments have low efficacy on this pathology.

Recently, neurophysiological and imaging studies allowed the identification of structural and functional cerebral abnormalities in brain areas and networks involved in pain processing and control. In this regard, particular attention has been paid to the application of non-invasive techniques, such as transcranial magnetic and electrical stimulation, able to stimulate the brain motor and prefrontal cortex and are reported in the literature as potentially valuable tools for the treatment of chronic pain [1]. 

Although Transcranial Direct Current Stimulation (TDCS) is still an experimental form of brain stimulation and is not a Food and Drug Administration (FDA)-approved treatment, research has demonstrated clinical improvement in chronic pain patients undergoing TDCS [2]. TDCS is the most common electrical stimulation and is based on the assumption that a weak constant direct current applied to the scalp for several minutes (up to 30 min) polarizes the tissues. This procedure has the advantage of being easy and safe [3]. Since it is a widely used technique to modulate pain perception, it has discordant results: while some authors [4,5,6] reported an attenuation of pain perception in their studies, others [7,8] showed no benefit. This could be explained by confounding factors such as depression, anxiety, or other comorbidities that could influence the efficacy of the TDCS [9].

During the past two decades, there has been a pronounced increase in the number of published research studies that have employed fNIRS to measure brain metabolic activity activation. fNIRS measures brain oxygenation and hemodynamics by detecting changes in the concentration of oxygenated and deoxygenated hemoglobin in the surface layers of the human cortex depending on how much near-infrared radiation is absorbed. The fNIRS technique overcomes different limitations of other neuroimaging technologies allowing acquisitions in a multitude of settings and during a wide variety of different tests, such as motor, somatosensory, or cognitive tasks [10]. 

The combination of fNIRS and TDCS can provide some clues to better investigate the effects of TDCS on the brain. Preliminary studies on animals [11] and humans [12,13] have been conducted using the combination of these two techniques. Merzagora et al. [13] analyzed the anterior prefrontal cortex effects of TDCS using fNIRS measurement through a prefrontal sensor. The results showed that fNIRS successfully captured activation changes induced by TDCS stimulation. Khan et al. [12] analyzed changed hemodynamic examples in the sensorimotor cortex because of bi-hemispheric TDCS polarities and their relationship to muscle movement and motor task execution. In our previous studies, FM patients showed an impairment in movement speed during finger-tapping tasks, corresponding to a reduced metabolism of motor cortical networks, detected by means of fNIRS [14,15]. In this pilot study, we investigated metabolic activity in FM patients compared to controls during a finger tapping task in basal condition, sham condition, and under anodal TDCS on M1, in order to understand if a single stimulation session can recover the reduced metabolism expected in FM patients. We aimed to clarify the biological basis of single TDCS stimulation, to reinforce the theoretical basis of the effectiveness of multiple sessions of M1 stimulation in FM. 

## 2. Materials and Methods

### 2.1. Subjects

Twenty-two healthy controls and 54 consecutive patients with Fibromyalgia were selected at the Neurophysiopathology Unit of Bari Policlinico General Hospital. The FM patients were enrolled after their first access, before starting the pharmacological treatment. All of the patients had been previously evaluated in the Rheumatology Unit of our Hospital, where active rheumatological diseases were excluded. The inclusion criteria were the diagnosis of FM, in agreement with the American College of Rheumatology (ACR) [16]. Exclusion criteria, evaluated during the first visit, were: age outside the 18–60 range, any further disease of the central or peripheral nervous system, including established diagnosis for psychiatric diseases, diabetes, renal insufficiency, autoimmune diseases, malignant neoplasms, as well as the use of Central Nervous System (CNS)-acting drugs or opioid therapy or analgesic treatment in the last 48 h. Among control subjects, we did not admit those with symptoms compatible with any form of pain symptoms in the last 3 months, including primary headaches [17]. All the participants were right-handed according to the score of the Edinburgh Handedness Inventory. The experimental procedures were approved by the ethics committee of the General Polyclinic of Bari. The number of the Ethical approval committee was 336/12-Bari Policlinico General Hospital. All participants provided written informed consent. The demographic and clinical data of the selected participants are detailed in Table 1. The demographic and clinical data of the real and sham groups matched (Table 1), except for age because controls were younger than patients in both groups (*p* < 0.05). However, we controlled each statistical result for age effects.

### 2.2. fNIRS System

**Figure 1:**
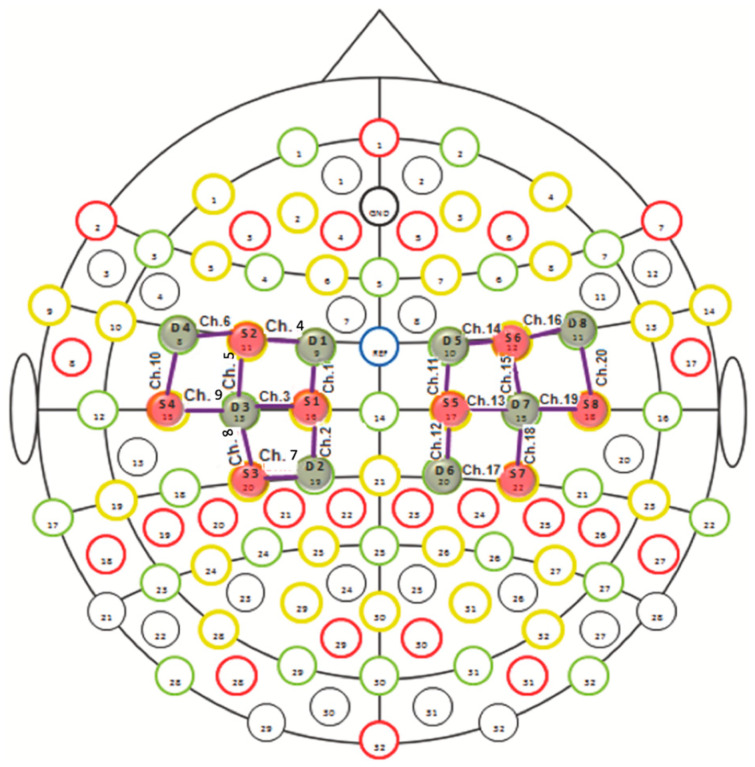
fNIRS monitoring with 20 channels in the motor cortical network. The red circles indicate sources and the blue circles represent detectors.

The study was performed by the continuous wave fNIRS system (NIRSport 8 × 8, Nirx Medical Technologies LLC, Berlin, Germany). The fNIRS device is a multi-channel system able to measure hemodynamic activity variations. For data acquisition, NIRStar 14.2 software was adopted (Version 14, Revision 2, Release Build, 2016-04-15 NIRx Medizintechnik GmbH, Berlin, Germany). The easy-to-use device involved LED sources and photosensitive detectors (sensitivity: >1 pW, dynamic range: >50 dB). The data were recorded by eight light sources and eight detectors. The lights were emitted from each source at two different wavelengths of 760 nm and 850 nm. The transmitter-receiver distance was 30 mm, as recommended in scientific literature. The location of sources, detectors, and the layout of the 20 fNIRS channels are illustrated in Figure 1. Specifically, the probes were placed on the cap over the primary and supplementary motor cortex. The sampling rate was 7.81 Hz. The optical density was converted to variations in oxyhemoglobin (HbO2) and deoxyhemoglobin (HbR), using the modified Beer–Lambert law [19]. Before each fNIRS recording, a calibration procedure was employed to determine the signal amplification required for every source-detector pattern.

### 2.3. Experimental Procedure

**Figure 2:**
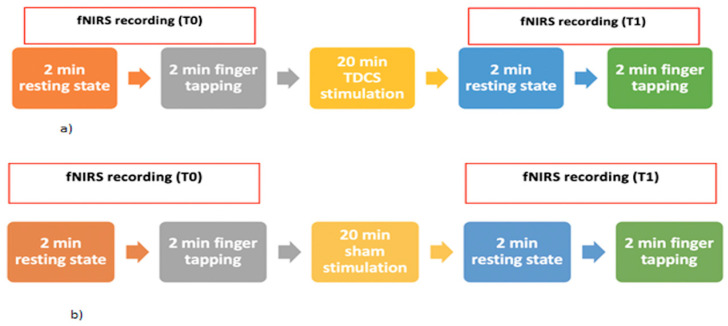
Experimental paradigm for the groups of the real (**a**) and sham (**b**) stimulation session using anodal TDCS.

The participants were seated on a comfortable chair in a quiet room and were informed of the experimental procedure. The participants were randomly assigned either to the real TDCS stimulation group or to the sham stimulation group. Both the real stimulation group and the sham stimulation group were told to receive the real TDCS. Eleven out of 22 normal controls and 28 out of 54 patients underwent a real TDCS whereas the other 11 normal controls and 26 patients underwent a sham stimulation (Figure 2).

#### Finger-Tapping Task Session

The technician placed the fNIRS recording cap over the participant’s head who stood still for two minutes (resting state) and then performed a finger-tapping task. The movement task consisted of pressing a push-button panel with the right-hand thumb as fast as possible. At the end of the session, the technician removed the fNIRS cap from the participant’s head and prepared them for the anodal TDCS stimulation session. After the TDCS stimulation, the technician and psychologist prepared the participant for a new recording session using fNIRS. Thus, the participants performed again the finger-tapping task for evaluating the possible changes in cerebral hemodynamic activity in the motor cortical regions.

### 2.4. TDCS Session Design

#### 2.4.1. Real Stimulation

A pair of surface sponge electrodes (35 cm^2^) were soaked in saline and applied to the scalp at the desired sites of stimulation in order to facilitate conductivity. Rubber bandages were used to hold the electrodes in place for the duration of stimulation. The subjects received left M1 stimulation by placing the anode on the scalp at C3 (International System 10–20) and the cathode on the right supraorbital region [20]. For the active TDCS condition, DC was delivered by a battery-driven constant current stimulator, with a maximum output of 10 mA. A constant current of 2 mA was applied for 20 min, as this intensity has been shown to be safe and painless.

#### 2.4.2. Sham Stimulation

The participants received simulated stimulation induced by placing the TDCS electrodes in the same position mentioned above but the stimulator was turned off after 30 s of stimulation being a reliable method of blinding.

### 2.5. fNIRS Processing

The fNIRS signal processing was performed using nirsLAB (version 2017.6) running on MATLAB (version R 2013 b). The quality of the signals was evaluated by checking that the gain factor (indicating how much photocurrent is amplified) and the coefficient of variation (the ratio between 100 times the standard deviation and the mean of the signal) were respectively lower than 8 and 7.5, two thresholds chosen during the calibration phase. If any channel did not pass the quality control, we attempted to improve the signal quality by making sure that the subjects’ hair would be kept away from the light path. First, signal processing was performed by removing discontinuities. Then, using the fNIRS artifact removal package of MNE (version 0.24.1), we automatically identified common types of artifacts in fNIRS data (spike and baseline shifts) and we removed them with the Remove Spike Artifacts GUI of nirsLAB. The raw data were filtered in the band-pass 0.004–0.1 Hz to remove low oscillations such as respiratory and cardiac frequencies from the fNIRS signal. The processed signals were then converted to optical intensities using the W. B Gratzer method [21] and the optical intensities in turn were converted to oxyhemoglobin and deoxyhemoglobin concentration changes using the modified Beer-Lambert law. Before computing the hemoglobin concentration changes, we carried out a baseline correction that was defined as the first 20 s of the total time of 120 s of resting state recorded before the finger-tapping task. In the present study, oxyhemoglobin levels are taken into account.

### 2.6. fNIRS and Behavioral Data Analysis

The finger-tapping speed was compared between patients and controls for the real and the sham stimulations, using the Student’s *t*-test for unpaired data. The fNIRS analysis focused on the recording during the finger-tapping task. To compute the degree of hemodynamic activation of each channel compared with the baseline, we used the Generalized linear model (GLM) implemented in nirsLab that, for the statistical analyses, relies on the Statistical Parameter Mapping 12 (SPM 12) tool. The GLM was carried out by choosing the Hemodynamic Response Function (HRF) to model the response during the finger-tapping task. Finally, the results obtained from the GLM were used to evaluate, using the Student’s *t*-test, if there were fNIRS channels wherein oxyhemoglobin changed in a statistically significant way (*p*-value < 0.05 corrected for multiple comparison) for the comparisons: patients vs. controls before and after the real stimulation, patients vs. controls before and after the sham stimulation. In addition, we evaluated, using the Pearson correlation test, if there was a statistical association between oxyhemoglobin levels in the significant channels respectively at T0 and T1 and the different clinical scores. The fNIRS and behavioral data are available on request.

## 3. Results

### 3.1. Finger Tapping Speed

**Figure 3:**
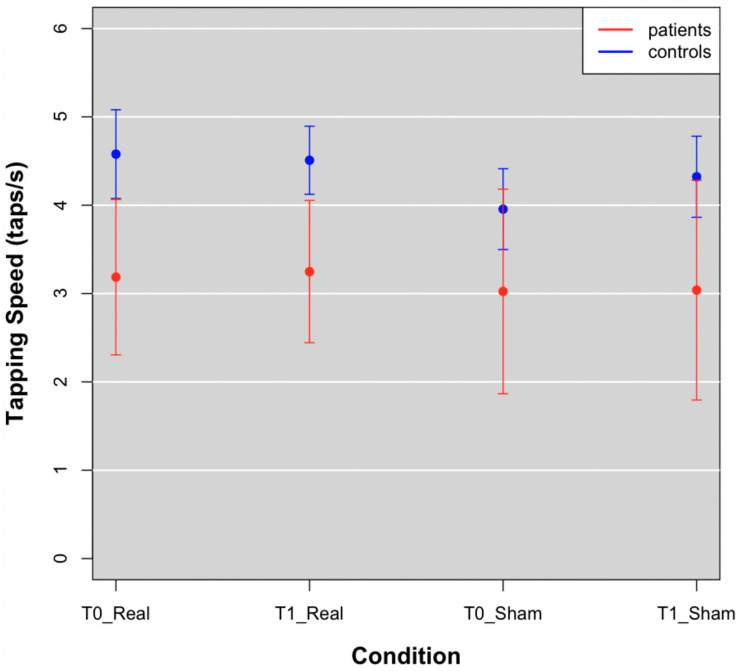
Tapping speed means of patients (red points) and controls (blue points) at T0 and T1 in Real and Sham conditions.

Movement speed was reduced in FM patients as compared to controls, at T0 and T1 in both real and sham groups (Figure 3; Table 2).

### 3.2. fNIRS Data

#### 3.2.1. Sham Stimulation

**Figure 4:**
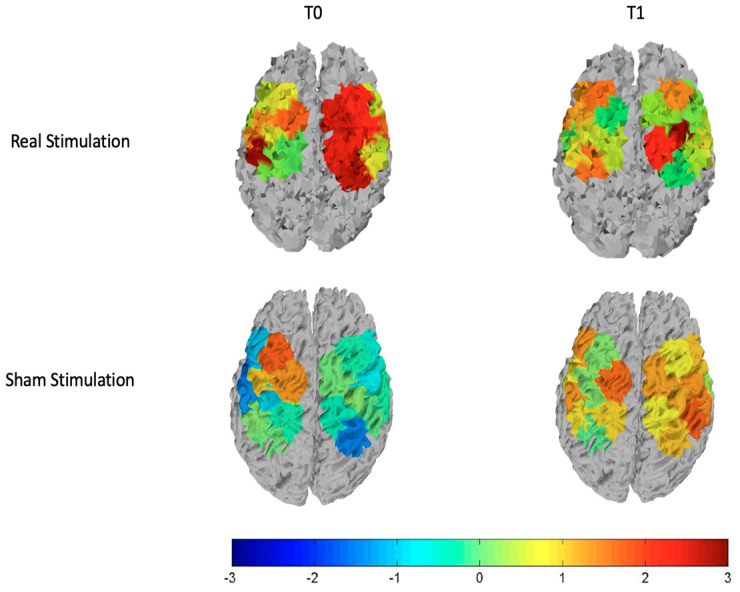
T-statistic maps of all the cortical motor regions for the real stimulation (**top** row) and the sham stimulation (**bottom** row). The left column is relative to the comparison controls vs. patients at T0 and the right column is relative to the comparison controls vs. patients at T1. Red and brown color expresses significant oxyhemoglobin reduction in fibromyalgic patients evaluated with an unpaired Student’s *t*-test. The scale refers to t values.

For the subjects who received sham stimulation, we observed that FM patients had reduced oxyhemoglobin levels in the basal condition, which did not change after the sham stimulation. The sham stimulation seemed to emphasize significant differences in oxyhemoglobin levels between groups, for an enhancing metabolic effect in controls, present on both the stimulated and not stimulated cortex (Table 3; Figure 4).

#### 3.2.2. Real Stimulation

**Figure 5:**
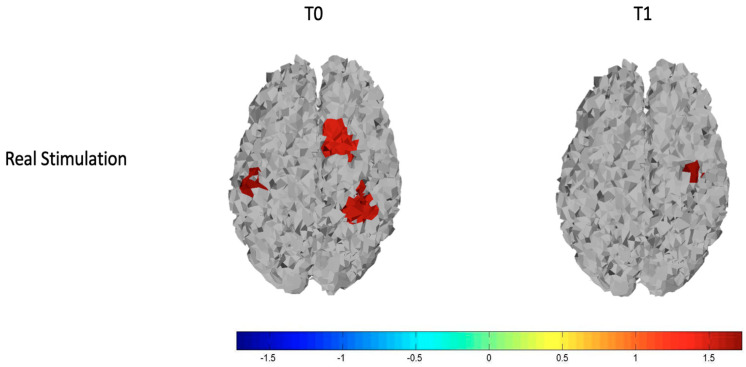
T-statistic map of the brain regions wherein there are significant differences in oxyhemoglobin for the comparisons of controls vs. patients at T0 (on the **left**) and controls vs. patients at T1 (on the **right**). Red and brown colors express significant oxyhemoglobin increase in controls evaluated with an unpaired Student’s *t*-test. The scale refers to t values.

We observed that basically patients had reduced cortical metabolism in both left and right hemispheres. After the stimulation, these differences disappeared on the stimulated hemisphere, and greater oxyhemoglobin level remained only in one channel of the right hemisphere, as we can see from the top row of Figure 4 and Figure 5 that display respectively the T-statistic map of all the cortical motor regions analyzed and the T-statistic map only of the regions wherein significant differences in oxyhemoglobin persist. (Figure 4 and Figure 5).

**Figure 6:**
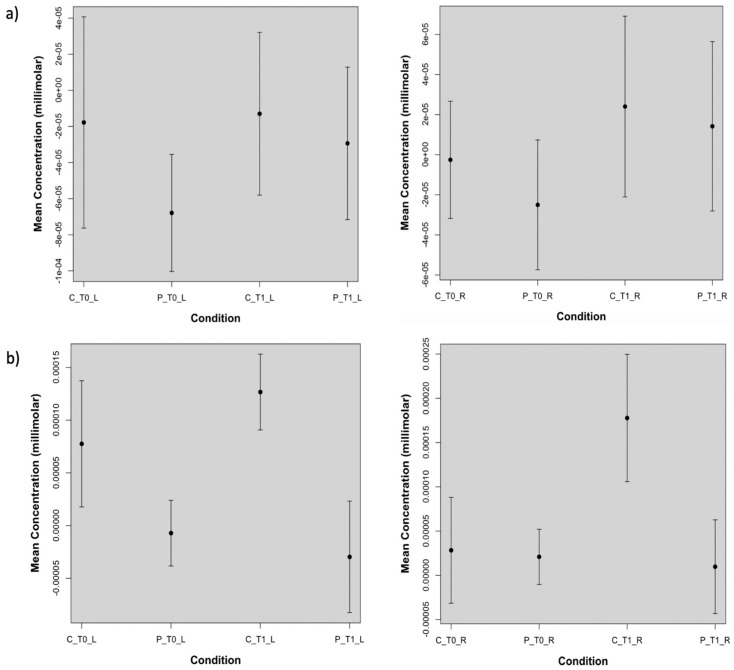
Plots of the oxyhemoglobin concentrations obtained from: (**a**) the real stimulation and averaged over the channels corresponding to the stimulated side and not stimulated side. On the left hemisphere, indicated with _L, real TDCS increased significantly (*p*-value lower than 0.05) oxyhemoglobin levels in patients (P), but not in controls. On the right hemisphere (_R) there was a significant oxyhemoglobin increase in controls (C) and in patients with *p*-values lower than 0.05 and 0.001, respectively. In (**b**) the sham effect is shown. On the stimulated and not stimulated hemisphere, the sham session determined an oxyhemoglobin increase in controls which is significant for the not stimulated side, leaving the oxy-hemoglobin levels in patients unchanged at T1.

We also evaluated, for both the real and sham stimulations, oxyhemoglobin levels averaged over the left and the right channels, corresponding to the stimulated and not stimulated hemispheres, respectively. This assessment confirmed that on the left hemisphere the real TDCS increased significantly (*p*-value < 0.05) cortical metabolism only in patients, while on the right hemisphere this effect was present in both groups but it was more evident in FM patients (Table 3). For the sham stimulation, controls showed a metabolic increase on both the stimulated and not stimulated hemisphere, while this increasing effect was absent in patients. (Figure 6, Table 3). In the sham experiment, significant differences in oxyhemoglobin levels between patients and controls on the left hemisphere persisted after the placebo stimulation (Figure 6, Table 3). For the different comparisons that we performed, we found a statistical power of 83 ± 14 (mean ± standard deviation) with Cohen’s effect size greater than 1.

We did not find any statistical association between the clinical and demographic data and the mean oxyhemoglobin concentration at T0 and T1, respectively.

## 4. Discussion

Although TDCS has been often reported as beneficial for FM management, evidence of its effectiveness is not robust enough and most studies on TDCS efficacy have used self-reported pain levels as the main outcome variable [22]. In particular, the demonstration of the anodal M1 TDCS efficacy in reducing FM pain is still open [23,24].

To the best of our knowledge, our study is the first to investigate TDCS effects on FM patients’ hemodynamic activity by performing an fNIRS analysis and a comparison with a control group. Our results show that after the stimulation most of the significant metabolic differences between patients and controls disappear. However, no effect on motor performance in the finger-tapping task was present. In the next paragraph, we report a detailed discussion on real and sham stimulation results.

### 4.1. Real Stimulation

On the stimulated hemisphere, we found that the reduction of cortical metabolism basically presents in FM patients, reverted after TDCS, while on the contralateral side, the significant oxyhemoglobin reduction persisted in a small region. Few reports are available about the neural and hemodynamic responses to anodal TDCS through joint imaging with EEG and fNIRS [25]. Studies investigating normal controls and post-stroke patients during resting state recorded with fNIRS before and after M1 anodal stimulation [26,27] showed that in both controls and patients, there was an increased metabolic activity of the motor network, which just confirmed that fNIRS could be sensitive to short term functional cortical changes. 

In this study, the anodal TDCS was applied after motor cortex activation by means of a finger-tapping task. Thus, our results suggest that in control individuals, who exhibited higher—normal-oxyhemoglobin levels with respect to patients, the lack of increasing effect in the stimulated hemisphere, observed in patients after real TDCS, was probably due to a ceiling effect. Transient inhibition could in fact occur in some situations of cortical hyper-activation (for example after active exercise) [28]. On the not stimulated hemisphere, controls exhibited an increased hemodynamic response after TDCS, which could be due to a minor effect of inhibitory control on the not previously activated cortex, or to the manifestation of a sham effect (see below), which did not imply a compensatory phenomenon of inhibition. 

In FM patients, who before TDCS expressed reduced cortical metabolism during active hand movement on both hemispheres, in accordance with previous studies [14,15], the anodal stimulation of M1 could compensate for this functional gap, restoring the activity at least on the stimulated hemisphere. On the opposite side, slight differences persisted between patients and controls, for a reduced effect in patients and the already explained phenomenon of increased oxyhemoglobin levels in controls.

The extension of the electrical field induced by TDCS is quite variable among subjects [29], so we cannot exclude an effect on the not stimulated hemisphere, which in any case was not enough to completely resolve the hemodynamic differences between patients and controls.

A number of studies evaluating hemodynamic activity in FM patients have reported that motor cortex metabolism is basically reduced when strictly related to active movement and that this reduction could be intrinsic to FM and contribute to poor control of pain [30].

These results may confirm that a single session of TDCS could be effective in restoring the reduced cortical metabolism of motor networks, which may be intrinsic to FM pathogenesis. 

### 4.2. SHAM Stimulation

Before and after the sham stimulation, no significant changes were found. However, we observed that on average, the sham stimulation produces higher hemodynamic activity in controls, especially on the not stimulated side. This suggests that, although in general patients benefit from placebo treatment to a greater degree than healthy individuals [22], from the hemodynamic point of view controls are more responsive to a possible placebo effect, in agreement with Monden et al. [31] wherein oxyhemoglobin signal in control was significantly higher than in post-placebo patients. Even though a placebo could act on clinical outcomes, the hemodynamic activity is not changed, at least in FM patients who show a cortical hypometabolism. 

We observed no effect on motor performance during the finger-tapping task in patients and controls after the real and sham stimulation. According to previous reports [14], FM patients had slower movement as compared to controls. The low motor efficiency, corresponded to a reduced metabolic activation of the cortical motor network, as also previously shown [14,15]. The motor impairment could be a tract of FM, due to an inhibitory effect on the motor cortex caused by chronic pain [32], or to a primary cortical motor network dysfunction, with a low efficient modulatory effect on noxious information processing. The lack of correlation between cortical hypometabolism and the severity of the disease, is a confirmation that hypometabolism is intrinsic to FM, rather than a consequence of a long chronic pain history. A single TDCS session, while partly resolving the cortical hypometabolism, was not able to act on the motor deficit in FM patients. This is in agreement with previous reports excluding a linear correlation between motor cortex oxyhemoglobin levels and finger tapping speed in FM patients and controls [14]. 

### 4.3. Limitations

We excluded control subjects with any form of ongoing pain or primary headaches; thus, this reduced the sample and caused the inclusion of younger people than the FM patients. Though we corrected all the statistics for age, this could not completely resolve the bias of possible age-related metabolic features. The different effects of real and sham stimulation in resolving the gap between patients and controls, could in any case indicate the direction of metabolic changes induced by a single session of TDCS in FM patients. 

## 5. Conclusions

This study provides evidence that a single session of anodal TDCS of the cortical motor network is able to restore basic cortical hypometabolism in FM patients. We did not establish the duration of this effect, or the effect of multiple sessions, but these results could support functional changes within the motor network. The question about the real significance of these metabolic changes in such complex disease and about the efficacy in restoring the motor network modulatory activity, could be resolved by studies exploring the long-term effect of M1 stimulation on cortical metabolism, pain processing, and clinical features in FM patients.

## Figures and Tables

**Table 1:** Demographic and clinical data for patients and controls that were selected for the real and the sham stimulation. All the data except the subjects’ gender are reported in terms of mean and standard deviation. P stands for patients, C for controls, WPI for Widespread Pain Index [16], SS for Symptom Severity Score [16], and FIQ for Fibromyalgia Impact Questionnaire [18].

Stimulation Type	Real		Sham	
Group	P (28)	C (11)	P (26)	C (11)
Age (y)	52.96 (±11.36)	33.64 (±12.99)	48.5 (±9.15)	37.36 (±15.4)
Sex (f/m)	21/2	9/2	20/2	7/4
Disease (y)	5 (±4.34)	-	7.73 (±6.32)	-
WPI	12.76 (±4.61)	-	13.95 (±4.35)	-
SS	7 (±2.34)	-	6.5 (±1.71)	-
FIQ	73.46 (±16.16)	-	64.72 (±12.27)	-

**Table 2:** Student’s *t*-test results reporting statistical differences of tapping speed between patients and controls.

Session	Comparison	T-Statistic	*p*-Value
Real	C_T0 vs. P_T0	5.9371 ***	<0.0001
Real	C_T1 vs. P_T1	6.3203 ***	<0.0001
Real	C_T0 vs. C_T1	0.34538	0.7341
Real	P_t0 vs. P_T1	−0.2658	0.7915
Sham	C_T0 vs. P_T0	3.0102 **	0.0065
Sham	C_T1 vs. P_T1	3.9529 ***	0.0006
Sham	C_T0 vs. C_T1	−1.3862	0.1958
Sham	P_T0 vs. P_T1	−0.0408	0.9677

Note. ** *p* < 0.01, *** *p* < 0.001.

**Table 3:** Student’s *t*-test results reporting statistical differences of oxyhemoglobin levels between patients and controls, referring to the averaged right (_R) and left (_L) channels. We controlled each statistical result for the effects of age by performing a regression test to see if the differences in hemoglobin concentration between the two groups were dependent on the subjects’ age.

Session	Comparison	T-Statistic	*p*-Value
REAL	C_T0_L vs. P_T0_L ***	4.0133	0.0001
REAL	C_T1_L vs. P_T1_L	0.9734	0.3325
REAL	C_T0_L vs. C_T1_L	−0.5403	0.5893
REAL	P_T0_L vs. P_T1_L *	−2.5246	0.0125
REAL	C_T0_R vs. P_T0_R ***	3.1521	0.0002
REAL	C_T1_L vs. P_T1_R	0.8633	0.4134
REAL	C_T0_R vs. C_T1_R *	−2.7332	0.0113
REAL	P_T0_R vs. P_T1_R ***	−4.6638	<0.0001
SHAM	C_T0_L vs. P_T0_L **	2.9672	0.0041
SHAM	C_T1_L vs. P_T1_L ***	4.7521	<0.0001
SHAM	C_T0_L vs. C_T1_L	−1.3811	0.1689
SHAM	P_T0_L vs. P_T1_L	0.6015	0.5479
SHAM	C_T0_R vs. P_T0_R	0.8598	0.3916
SHAM	C_T1_R vs. P_T1_R ***	4.9708	<0.0001
SHAM	C_T0_R vs. C_T1_R ***	−4.9929	<0.0001
SHAM	P_T0_R vs. P_T1_R	1.2743	0.2041

Note. * *p* < 0.05, ** *p* < 0.01, *** *p* < 0.001.

## Data Availability

The data presented in this study are available on request from the corresponding author. The data are not publicly available due to privacy restrictions as formally.
